# Autologous Microfragmented Adipose Tissue for the Treatment of Knee Osteoarthritis: Real-World Data at Two Years Follow-Up

**DOI:** 10.3390/jcm11051268

**Published:** 2022-02-25

**Authors:** Daniele Screpis, Simone Natali, Luca Farinelli, Gianluca Piovan, Venanzio Iacono, Laura de Girolamo, Marco Viganò, Claudio Zorzi

**Affiliations:** 1 Department of Orthopaedics, IRCCS Ospedale Sacro Cuore Don Calabria, 37024 Negrar, Italy; daniele.screpis@sacrocuore.it (D.S.); gianluca.piovan@sacrocuore.it (G.P.); venanzio.iacono@sacrocuore.it (V.I.); claudio.zorzi@sacrocuore.it (C.Z.); 2 Clinical Ortopaedics, Department of Clinical and Molecular Sciences, Università Politecnica delle Marche, 60020 Ancona, Italy; farinelli.luca92@gmail.com; 3 Orthopaedic Biotechnology Laboratory, IRCCS Istituto Ortopedico Galeazzi, 20161 Milan, Italy; laura.degirolamo@grupposandonato.it (L.d.G.); marco.vigano@grupposandonato.it (M.V.)

**Keywords:** osteoarthritis, autologous microfragment adipose tissue, orthobiologics, knee, adipose-derived mesenchymal stem cells

## Abstract

The purpose of the present study was to assess, prospectively, the safety, clinical effectiveness, and feasibility of a single intra-articular injection of microfragmented adipose tissue in different stages of knee osteoarthritis (OA). The study included patients (aged 18–70 years), affected by OA (Kellgren–Lawrence I-IV). Unselected patients were evaluated before and prospectively after 6, 12, and 24 months from the injection. Visual analog scale (VAS) and knee injury and osteoarthritis outcome score (KOOS) were used for clinical evaluations. A total of 202 patients were eligible. The mean follow-up time in the cohort of patients was 24.5 ± 9.6 months. Total KOOS significantly improved from pre-operative baseline levels to 6-month follow-up (*p* < 0.001), and again between 6- and 12-month follow-ups (*p* < 0.001). The VAS showed a prompt reduction at 6 months (*p* < 0.001 vs. baseline), but then it increased again at 12 months compared to the 6-month assessment (*p* < 0.001), even though it remained lower than baseline (*p* < 0.001). At 24 months, patients with KL-IV demonstrated a lower improvement compared to baseline; patients that had undergone previous corticosteroid injections had a greater risk to further injection treatment. The collected clinical results suggest that MFAT may represent a safe and effective treatment for OA symptoms, offering a low-demanding and minimally invasive treatment.

## 1. Introduction

Osteoarthritis (OA) is a chronic progressive joint disease, and is one of the leading causes of disability. Recently, there has been great interest about biological procedures in early degenerative disease. The treatment by intra-articular injection of adipose-derived mesenchymal stem cells (ASC) represents an innovative and promising approach for knee and ankle OA [1,2,3,4,5]. In fact, both in vitro and in vivo studies clearly demonstrated anti-inflammatory and regenerative properties of ASC in cartilage repair [6].

ASC are traditionally obtained after enzymatic digestion and prolonged expansion in vitro; however, their use is strictly regulated by complicated legislation. Therefore, in the last years, several strategies have been proposed to exploit the regenerative properties of adipose tissue cells through mechanical/non-enzymatic methods, and without expansion, resulting in the development of minimally manipulated products [7]. Among them, microfragmented adipose tissue (MFAT) has been referred to as one of the smartest and easiest ways to use adipose tissue in regenerative procedures in a variety of clinical applications [3,5,8]. Following this approach, the adipose tissue generally harvested through liposuction is processed by means of a washing and resizing device [9,10]. Throughout this process, mild mechanical forces are applied, resulting in washed, emulsified, and microfragmented adipose tissue, with removal of blood and oil residues. To date, several studies have been published reporting promising results in the treatment of knee OA by autologous microfragmented adipose tissue injection [11].

The purpose of the present study was to assess, prospectively, the safety, clinical effectiveness, and feasibility of a single intra-articular injection of microfragmented adipose tissue in different stages of knee OA, with particular focus on the improvement of symptoms, and the delay for further treatments (injection or surgery). This prospective study was performed on an unselected cohort, in order to collect “real-world” data, which are representative of the outcomes of this procedure in actual clinical practice. Indeed, these data allow us to determine Real-World Evidence, which nowadays represents an important aspect in the evaluation of treatment safety and effectiveness [12].

## 2. Materials and Methods

### 2.1. Study Design and Population

This prospective open-label, single-center, uncontrolled study was conducted with the highest respect for individual participants. The procedures followed were in accordance with the ethical standards of the responsible committee on human experimentation (institutional and national), and with the revision of the Declaration of Helsinki, 2014. Before the beginning of any study-related activities, each study participant signed informed consent. The present study was approved by the Ethics Committee of Verona and Rovigo, Italy (protocol n. 61386–19 September 2018). Patients were recruited from January 2018 to January 2019.

The study included patients who were between 18 and 70 years old, affected by degenerative joint changes (Kellgren–Lawrence I-IV), with a history of failed previous conservative treatment (anti-inflammatory, physical therapy, intra-articular steroid, and/or viscosupplementation, and/or platelet-rich plasma (PRP)) and chronic pain ≥4 months with limitation of daily activities. Exclusion criteria included: (1) intra-articular steroid or viscosupplementation injections performed within the last 3 months; (2) concomitant severe infection, malignant tumor, coagulation disorder, or uncontrolled or unmanageable systemic diseases; (3) rheumatic diseases; (4) coronal malalignment of lower limb (hip–knee-angle >190° and <170°).

Patients were evaluated before the treatment, and prospectively after 6, 12, and 24 months from the injection. Visual analog scale (VAS) and knee injury and osteoarthritis outcome score (KOOS) were used for clinical evaluations. Adverse events and further treatments were recorded. Baseline characteristics, habits, and comorbidities were recorded before the injection.

### 2.2. Harvesting the Adipose Tissue

The patient was placed in a supine position. After local anesthesia, a small incision was made to insert a 17G blunt cannula (connected to a Luer-lock 60-cc syringe), and Klein sterile solution (containing saline, lignocaine, and epinephrine) was injected into the subcutaneous fat. Approximately 150–200 mL of this solution was injected in 50-mL aliquots into the lower abdominal area. Adipose tissue (approximately 50 mL) was then harvested manually via a 13G blunt cannula connected to the syringe. The area of fat harvest was tailored to the body habitus of each patient (normally lower abdomen or flank areas).

### 2.3. Processing the Lipoaspirate and Injecting the Microfragmented Adipose Tissue

The lipoaspirate was processed in a closed and aseptic manner using the Lipogems^®^ system following the manufacturer’s instruction [13,14], and according to a previously published technique [13]. The resulting product was then filtered through a 500-μm micron filter to obtain the microfragmented adipose tissue. Before injecting, the skin was sterilely dressed, and the injection was performed on the suprapatellar pouch with a superolateral approach. All of the patients received 8 mL of microfragmented adipose tissue. An abdominal binder was then applied to the adipose tissue harvest site for two weeks. In case of knee pain during the treatment, they were recommended to use cold therapy, and to rest for at least 24 h. Patients were allowed to take acetaminophen in case of pain. Non-steroidal anti-inflammatory drugs (NSAIDs) were not allowed. Otherwise, mild activities and a gradual resumption of normal sport or recreational activities were allowed as tolerated.

### 2.4. Statistical Analysis

The analyses were performed using R software v4.1.1 (R Core Team, Wien, Austria). A Shapiro–Wilk test was used to assess data distribution for each continuous variable, and data reporting and analyses were performed accordingly to the result of this test. Associations were evaluated by multiple linear or logistic regression models selected using the BIC criterion using multi package for R [11], depending on the nature of the dependent variable. The following groups of covariates were considered: demographics (age, sex, BMI, sport activity, smoke habits), comorbidities (heart disease, thyroid disease, diabetes, hypertension), diagnosis (OA, chondropathy, early OA, meniscal lesions, ligament lesions, tendinopathy, bone edema, KL grade), previous surgeries (ACL reconstruction, meniscal repair, osteotomy, arthroscopy), previous injections (with viscosupplementation, PRP, MFAT, NSAIDs, corticosteroids), use of physical therapy (before and after injection). For the evaluation of the effects of these variables on score changes, the baseline score was added to the model as an independent variable together with the follow-up time, in order to control for these parameters. Comparisons among different time points were performed using one-way ANOVA or a Kruskal–Wallis test with appropriate post hoc test. In the presence of two independent variables, two-way ANOVA models with Tukey’s post hoc test were used to measure the influence of each variable and their interaction. A Chi-squared test or Fisher’s exact test (when applicable) were used to evaluate different distributions of categorical variables between or among subgroups. Values of *p* < 0.05 were considered statistically significant.

## 3. Results

### 3.1. Patients’ Demographic

A total of 202 patients were eligible for analysis (105 females, 97 males) with a mean age of 54.0 ± 9.0 years old. The mean follow-up time in the cohort of patients was 24.5 ± 9.6 months. Other demographic characteristics are reported in Table 1. Comorbidities were present in 79 cases, mostly represented by hypertension (*n* = 46), thyroid diseases (*n* = 23), and diabetes (*n* = 10). Previous surgeries were performed on 53.9% of patients, with the most frequent being arthroscopy for cartilage lesion (*n* = 60), followed by meniscus surgery (*n* = 52), and ACL reconstruction (*n* = 26). Among the patients, 48.0% of them underwent injections before MFAT treatment. The most frequent treatment was hyaluronic acid (89.7%), followed by platelet-rich plasma (PRP) (19.6%), steroids (18.6%), NSAIDs (3.1%), and MFAT (2.1%). Among the patients, 33.1% of them underwent previous injections with two different products.

### 3.2. Patient-Reported Outcome Measures

**Figure 1:**
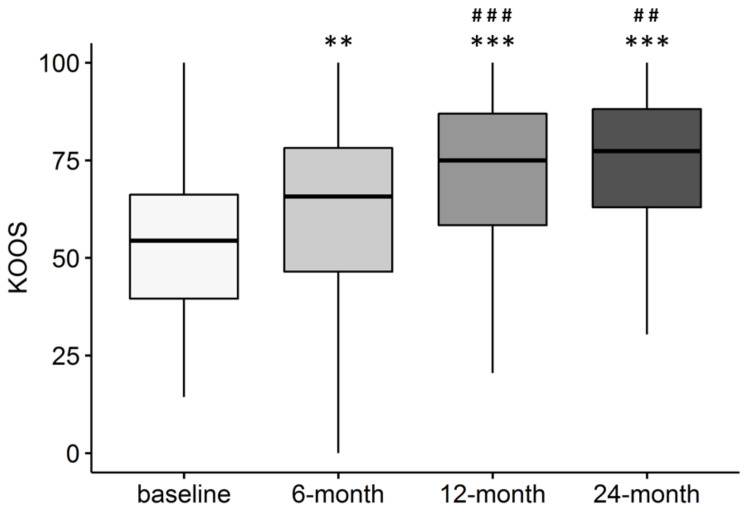
Total KOOS score over time in patients treated with intra-articular injection of MFAT. ** *p* < 0.01; *** *p* < 0.001 vs. baseline; ## *p* < 0.01; ### *p* < 0.001 vs. 6-month follow-up. Pre: *n* = 202; 6 month: *n* = 189; 12 month: *n* = 181; 24 month: *n* = 150.

Total KOOS significantly improved from baseline to 6-month follow-up (*p* < 0.001), and again between 6- and 12-month follow-ups (*p* < 0.001). At 24 months, improvement was significant compared to baseline (*p* < 0.001) and 6-month follow-up (*p* = 0.002), but it was similar to the score recorded 12 months after injection (*p* = n.s.) (Figure 1).

No difference with respect to total KOOS score was found by analyzing the single KOOS subscales: pain, activity and daily living, symptoms, sport, and quality of life.

**Figure 2:**
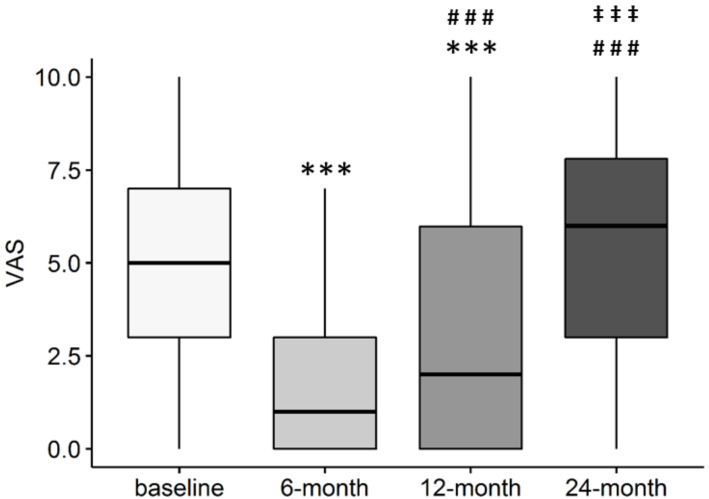
VAS score over time in patients treated with intra-articular injection of MFAT. *** *p* < 0.001 vs. baseline; ### *p* < 0.001 vs. 6-month follow-up; ^‡‡‡^ *p* < 0.001 vs. 12-month follow-up. pre: *n* = 202; 6 month: *n* = 189; 12 month: *n* = 181; 24 month: *n* = 150.

On the contrary, VAS showed a prompt reduction at 6 months (*p* < 0.001 vs. baseline), but then it increased again at 12 months compared to the 6-month assessment (*p* < 0.001), even though it remained lower than baseline (*p* < 0.001). At 24 months after injection, VAS was similar to baseline values (*p* = n.s), and significantly higher than 6- and 12-month follow-ups (*p* < 0.001 both) (Figure 2).

Nevertheless, these results were not confirmed by KOOS pain subscales, where significant improvements compared to baseline were observed at all time points (Table 2). The two methods for the measurement of pain are different, with VAS reporting an overall pain intensity, whereas the KOOS pain subscale takes into account several aspects, such as frequency, and activity-related pain perception.

Overall, patients were largely satisfied at the last follow-up, with 64.5% of patients reporting “Very good” or “Good” satisfaction, and only 20.3% of patients declaring a “Poor” or “Very poor” satisfaction.

### 3.3. Variation of KOOS and VAS Depending on OA KL Grade

**Figure 3:**
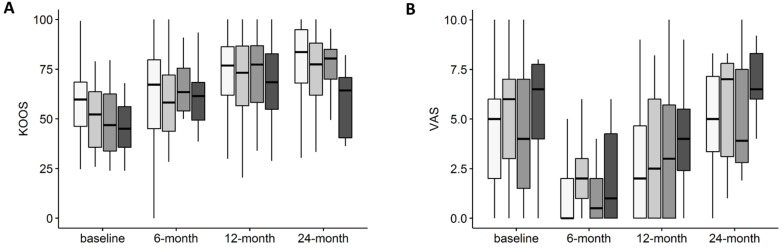
(**A**) Total KOOS score over time in patients with different OA KL grade treated with intra-articular injection of MFAT. (**B**) VAS score over time in patients with different OA KL grade treated with intra-articular injection of MFAT. KL1 *n* = 80, KL2 *n* = 46, KL3 *n* = 32, KL4 *n* = 19.

Data about KL grade were available for 177 subjects. KOOS improvements over time were independent from KL grade (interaction: *p* = n.s.). The effects of both time (*p* < 0.001) and KL grade (*p* = 0.012) alone were significant (Figure 3A). Despite the lack of significance, at 24 months, patients with KL4 demonstrated a lower improvement compared to baseline; this observation may be possibly suggestive of a reduced efficacy of the treatment in this category of patients, especially considering the reduced numbers of the subgroup analysis (only seven patients with KL4 responded at 24-month follow-up). Likewise, VAS improvements were also similar in patients affected by different KL grade OA (interaction: *p* = n.s.) (Figure 3B). The effect of time alone has a significant impact on VAS (*p* < 0.001), as well as the effect of OA severity alone (*p* = 0.004). Values are reported in Table S1.

### 3.4. Objective Outcomes

The frequency of new injections in the cohort during the evaluation period was 16.3% (*n* = 33), whereas 5.9% of the patients underwent surgeries (*n* = 12), seven of which were joint replacements (3.4%). New injections were reported between 11.8 and 39.9 months after treatment (median 26.8 months), whereas surgeries were reported between 17.5 and 40.0 months after MFAT injections (median 25.9), with knee replacement specifically at a median follow-up of 34.3 months.

Among the 74 patients who were using analgesic drugs before the MFAT treatment, at 12-month follow-up, 45.9% of them (*n* = 34) discontinued their use. On the other hand, 17 patients declared to have started using painkillers after the treatment (13.2%). Among those who continued to use them, the frequency of analgesic drug consumption dropped from eight times per month at baseline (IQR 4–30) to four times per month (2–8) at 12-month follow-up (*p* = n.s.).

### 3.5. Association of Patients’ Characteristics with Subjective and Objective Outcomes

Considering KOOS value at the last follow-up, thyroid disease significantly associated with worse results (*p* = 0.003), as did diabetes, though in a non-significant manner (*p* = 0.064). Other comorbidities did not significantly influence the outcome. When adjusted for other diagnostic parameters, KL4 was significantly associated with a lower KOOS score at the last follow-up (*p* = 0.043). Interestingly, the last KOOS score was also negatively influenced by physical therapy after the injection (*p* = 0.042). These patients showed an adjusted mean of −6.6 ± 3.2 points compared to patients who did not undergo physical therapy. Conversely, previous ACL reconstruction surgery was associated with better KOOS score at the last follow-up (*p* = 0.013).

Concerning VAS score at the last follow-up, lower pain scores were associated with previous PRP injections (*n* = 17), once adjusted for baseline pain score and follow-up time (Table 3). No association with VAS or KOOS values at final follow-up were observed concerning demographic characteristics.

No significant associations were observed between treatment satisfaction and patients’ characteristics such as diagnosis, comorbidities, and previous interventions.

Considering objective outcomes, the event “further injection treatments” significantly associated with previous corticosteroid injections (*p* = 0.003). In particular, the odds ratio of undergoing further injections after MFAT treatment was 4.8 in patients who underwent previous corticosteroids injections compared to those who did not. In addition, post-treatment physical therapy was associated with additional injections (*p* = 0.036), whereas no association was observed between physical therapy and further surgeries.

New surgeries were associated with hypertension (odds ratio of 3.8 in those who had it compared to those who did not, *p* = 0.038), whereas a negative association for further surgeries was observed for those who practiced more sport in terms of hours per week (*p* = 0.051, 2.9 ± 3.3 h/week vs. 0.7 ± 1.8 h/week for patients who received further surgeries and who did not, respectively). No significant associations were observed for the odds of undergoing a knee replacement.

A higher BMI was associated with more frequent painkiller consumption at 12 months (*p* = 0.023).

## 4. Discussion

The main finding of the present study is that a single autologous MFAT injection represents an effective treatment for knee OA, regardless of OA KL grade. Specifically, 34.2% of patients reported a >50% improvement of KOOS compared to pre-treatment values at a mean follow-up of 24.5 months. Moreover, 26.9% of patients reported a >50% decrease in VAS values compared to pre-treatment values. No adverse events were observed in the cohort, confirming the safety profile of this procedure.

This study population was heterogeneous, as it represented the real-world patients, and, therefore, allowed for the search of possible associations between clinical outcomes and patients’ characteristics. No significant associations were observed between clinical results and patients’ characteristics, diagnosis, and previous interventions, with the exception of thyroid disorders and diabetes, which were associated with lower clinical results at the last follow-up, probably due to faster progression of OA [15,16].

Conversely, significant associations were found among some of the baseline patients’ characteristics and the odds of receiving additional treatments after MFAT injection, as well as the analgesic consumption. In particular, 52 patients (25.7%) underwent additional treatments during the study period, such as injective or surgical treatments. It is worth mentioning that most of the patients included in the study had undergone several previous treatments as surgery and/or injections before the MFAT treatment, since MFAT injection represents one of the last treatment options before joint replacement [8,17]. Interestingly, the patients that had undergone previous corticosteroid injections had a greater risk to further injection treatment. A growing body of evidence suggests that intra-articular corticosteroid injection can accelerate the progression of joint degeneration [18]. It is plausible that this progression of OA determines a greater risk of further injection for the patient. Analyzing our results, we reported that physical therapy after injection, and sport activity, were associated with lower clinical results and further treatments. These observations are suggestive of a growing need of the development of specific rehabilitation strategies aimed at supporting the functional recovery, and avoiding functional overload, after MFAT treatment.

Hypertension was positively associated with the odds of receiving further surgeries, whereas a negative association was found with weekly sport hours, with patients who practiced sport before receiving MFAT injection being less prone to receive surgical interventions.

Finally, a higher BMI was associated with more frequent painkiller consumption at 12 months. This observation was not surprising, since obesity is associated with a prolonged use of painkillers at 12-month follow-up even after total joint arthroplasty [19].

In analogy to several studies [20], we reported that pain and function scores improved for all of the KL grades of knee OA between baseline and 12- and 24-month follow-ups, even though VAS showed a different trend at 24 months. This observation is discordant with the results from the KOOS pain subscale, which improved significantly at 24 months in our study, as well as with the few available previous reports [21]. Though differences with other studies may be explained by the possible different characteristics of the study populations, the lack of consistency between the two subscales in capturing pain reduction was quite unanticipated. A possible explanation could be related to the fact that VAS was reported to be difficult to understand, especially when the question was asked without reference to a specific activity, leading to possible misunderstandings, and thus, reducing the effect size and reliability compared to the specific questions within the KOOS questionnaire [22]. In addition, it has been reported that a single VAS question is not interchangeable with multi-item Likert indices (as in the KOOS pain questionnaire), and thus, discrepancies are likely to happen between different measurement methods [23]. Generally, we observed that the improvement of KOOS score lasts until 24 months, even though the score tends to deteriorate after 12 months in cases of advance knee OA (KL-IV). Therefore, the procedure needs to be repeated.

The rationale behind the beneficial effects observed in this study resides on a number of basic sciences evidence, obtained in in vitro and preclinical trials. Indeed, MFAT was demonstrated to support cell viability, proliferation, and extracellular matrix deposition, as well as to reduce the markers of inflammation and matrix degradation in several cell types, including cartilage cells and synoviocytes [24]. In vivo, MFAT was able to improve cartilage defects repair [25], and to exert a chondro-protective action in a rabbit model of OA [26]. The mechanism of these actions depends on paracrine effectors, such as soluble molecules or extracellular vesicles that are released by the cells contained in MFAT, and that have been identified as mediators with anti-inflammatory and pro-regenerative properties [27,28].

The main limitation of this study is the absence of a control group, although it is unclear what a reference control group should be. The patients present all grades of OA, where those patients with a deformity greater than ten degrees were excluded. It can be argued that this represents a heterogeneous group of disease. Combining the age range of our cohort (18 to 70 y/old), as well as the severity of their conditions (KL grade I-IV), makes for many variables, and thus, makes subgroup analysis difficult. However, this is a pragmatic representation of our clinical practice, and the highly statistically significant improvement of pain, function, and quality of life cannot be ignored.

## 5. Conclusions

We reported the prospective results of a large, unselected cohort of patients. Although these findings need to be validated with a randomized controlled trial, these data represent Real-World Evidence, demonstrating that MFAT represents a safe and effective treatment for OA symptoms, offering a low-demanding and minimally invasive therapeutic option for patients with very different characteristics, who are not eligible for more invasive approaches, such as arthroplasty. Advanced OA (KL-IV) and comorbidities might be associated with worse clinical results. Moreover, further studies are needed to establish more clearly which stage of the disease and characteristics of the patient are best-suited for treatment with biologics to maximize patient-centered efficacy.

## Figures and Tables

**Table 1:** Patient demographics.

Characteristics	
Age (years)	54.0 ± 9.0
Gender (F/M)	105/97
BMI	26.8 ± 4.2
BMI category (Normal weight/Overweight/Obese)	70/94/38
Smokers (yes/former/no)	24/69/109
**Diagnosis**	
Monolateral/Bilateral	188/14
Treated for OA (yes/no)	154/48
Kellgren–Lawrence grade (0/1/2/3/4)	25/80/46/32/19
Comorbidities (yes/no)	77/125
Hypertension	46
Thyroid disorders	23
Diabetes	10
Arthritis	7
Cardiovascular disease	4
Fibromyalgia	1
**Sport**	
Sport Activity (None/Recreational/Competitive)	43/154/3
Hours/week sport activity *	3.53 ± 3.3
**Previous and Current Treatments/Surgeries at Baseline**	
Previous surgeries (yes/no)	109/93
Previous injections (yes/no)	97/105
In treatment with painkillers (yes/no)	74/128
Painkiller administrations per month	7.0 ± 9.9
In treatment with physical therapy (yes/no) **	115/87

* Only patients who practice sport (recreational or competitive). BMI: body mass index; OA: osteoarthritis. ** Refers to high intensity physical therapy. Data are expressed as absolute frequency or mean ± SD.

**Table 2:** KOOS pain score at different time points.

Follow-Up	KOOS Pain Score	*p* Value vs. Baseline
Baseline	63.9 (47.2–77.8)	-
6-month	80.6 (69.4–91.7)	*p* < 0.001
12-month	83.3 (66.7–93.7)	*p* < 0.001
24-month	83.3 (68.7–94.4)	*p* < 0.001

**Table 3:** Adjusted differences in KOOS and VAS scores at the last follow-up in patients with different characteristics.

	Change vs. Reference	*p* Value	Model Covariates
**KOOS**			
Thyroid disease	−14.4 ± 4.9	*p* = 0.003	Diabetes, KOOS at baseline
Diabetes	−12.5 ± 6.7	*p* = 0.064	Thyroid disease, KOOS at baseline
KL grade 4	−21.05 ± 10.3	*p* = 0.043	KOOS at baseline
Previous ACL reconstruction	+10.0 ± 4.0	*p* = 0.013	KOOS at baseline
**VAS**			
Previous PRP injections	−1.83 ± 0.70	*p* = 0.010	VAS at baseline, follow-up time

Adjusted changes were calculated using linear model selected for Bayesian information criteria (BIC) minimization. Several models were tested for the following categories of variables: demographics, comorbidities, previous intervention, and diagnosis. Reference is represented by mean of patients who did not present the specific condition.

## Data Availability

The data presented in this study are available on request from the corresponding author.

## Supplementary Materials

The following supporting information can be downloaded at: https://www.mdpi.com/article/10.3390/jcm11051268/s1, Table S1: Values of KOOS and VAS in patients with different KL grade OA during the study period.
