# Supplementary material for: Autologous Microfragmented Adipose Tissue for the Treatment of Knee Osteoarthritis: Real-World Data at Two Years Follow-Up
Source: J Clin Med. 2022 Feb 25;11(5):1268. doi: 10.3390/jcm11051268 (PMC8911134; doi:10.3390/jcm11051268)
Supplement: Supplementary file 1 [file jcm-11-01268-s001.zip › jcm-1581625-supplementary.pdf]

Supplementary Table S1. Values of KOOS and VAS in patients with different KL grade OA during the study period.

KOOS				
	Pre-operative	6 months	12 months	24 months
KL1	59.74 (46.14 - 68.46)	67.18 (44.99 - 79.69)	76.79 (61.9 - 86.31)	83.63 (68.01 - 94.79)
KL2	52.2 (35.62 - 63.63)	58.24 (43.71 - 72.02)	73.15 (56.57 - 86.61)	77.38 (61.9 - 88.1)
KL3	46.85 (33.74 - 62.44)	63.42 (54.01 - 75.47)	77.37 (58.26 - 86.78)	80.36 (69.94 - 84.97)
KL4	45.05 (35.67 - 56.14)	61.43 (49.26 - 68.34)	68.45 (54.79 - 82.74)	64.29 (40.48 - 70.83)
**VAS**				
	Pre-operative	6 months	12 months	24 months
KL1	5 (2 - 6)	0 (0 - 2)	2 (0 - 4.65)	5 (3.35 - 7.15)
KL2	6 (3 - 7)	2 (1 - 3)	2.5 (0 - 6)	7 (3.1 - 7.8)
KL3	4 (1.5 - 7)	0.5 (0 - 2)	3 (0 - 5.7)	3.9 (2.8 - 7.5)
KL4	6.5 (4 - 7.75)	1 (0 - 4.25)	4 (2.4 - 5.5)	6.5 (6 - 8.3)

Data reported as median (interquartile range). KL1 n=80, KL2 n=46, KL3 n=32, KL4 n=19.
